# Synthesis of Peptide Radiopharmaceuticals for the Therapy and Diagnosis of Tumor Diseases

**DOI:** 10.3390/molecules18033379

**Published:** 2013-03-14

**Authors:** Mazen Jamous, Uwe Haberkorn, Walter Mier

**Affiliations:** Department of Nuclear Medicine, University Hospital Heidelberg, Im Neuenheimer Feld 400, D-69120 Heidelberg, Germany

**Keywords:** radionuclides, chelator, prosthetic groups, carrier molecules, peptides, medicinal application, radiopharmaceutical, diagnostic imaging, radiotherapeutics

## Abstract

Despite the advances in molecular biology and biochemistry, the prognosis of patients suffering from tumor diseases remains poor. The limited therapeutic success can be explained by the insufficient performance of the common chemotherapeutic drugs that lack the ability to specifically target tumor tissues. Recently peptide radiopharmaceuticals have been developed that enable the concurrent imaging and therapy of tumors expressing a specific target. Here, with a special emphasis on the synthesis of the building blocks required for the complexation of metallic radioisotopes, the requirements to the design and synthesis of radiolabeled peptides for clinical applications are described.

## 1. Introduction

The incidence of human malignant tumor diseases is still increasing worldwide. Generally, cancer treatment can be performed using one or a combination of the following methods: surgery, chemotherapy and radiation therapy. Their side effects limit the efficiency of chemo- and radiotherapeutic agents, but can be avoided and a much more effective therapy is possible if the drugs used have tumor selectivity. This involves the determination of biochemical processes that distinguish tumor tissue samples from healthy tissue (Table 1). As a result, tumor-specific biomarkers are used in oncology. Several types of agents have been developed for specific accumulation in the malignant cells to reduce the cytotoxic effect on the normal cells. These agents can be labeled with radionuclides that accumulate in the tissue of interest. Depending on the purpose, gamma or positron emitters are used for diagnosis and beta, alpha or Auger electron emitters are used for therapeutic applications in cancer treatment. The higher the specific activity of a drug, the better the imaging and the lower the cytotoxic side-effects in therapeutic applications [1].

Modern imaging methods include computer tomography (CT), magnetic resonance tomography (MRI), ultrasound, single-photon emission computed tomography (SPECT) and positron emission tomography (PET). They provide information about the phenotypic functional changes associated with the development of the disease. New treatment modalities based on the biological properties of tissues have been developed, where important progress has been achieved using antibodies and peptides [2]. When labeled with therapeutic radioisotopes, these agents are suitable for endoradiotherapy and exploit their high specificity. This has been realized for antibodies against the tumor associated epitope CD20 [3] or peptides binding to the somatostatin receptors [4].

**Table 1:** Biomarkers used in clinical routine for tumor-diagnosis [5].

Perfusion	[^15^O]H_2_O
Glucose metabolism	[^18^F]FDG
Bone metabolism	[^18^F]Fluoride
Choline metabolism	[^18^F]Choline
DNA synthesis	[^18^F]FLT
Amino acid transport and protein synthesis	[^18^F]FET, [^11^C]MET, [^18^F]FDOPA
Receptor binding	[^68^Ga]-DOTA-TOC
Antigen binding	[^111^In]-anti-CD20 mAb
PSMA	[^68^Ga]-PSMA
Angiogenesis	[^18^F]Galacto-RGD
Lipid synthesis	[^11^C]AcOH
Hypoxia	[^18^F]FAZA, [^18^F]MISO
Apoptosis	[^124^I]Annexin V
Gene expression	[^18^F]FHBG

Many specific radiopharmaceuticals have been developed in various preclinical and clinical stages for imaging and therapy of tumor diseases and some of them are currently in routine clinical use. They can be classified into three major categories according to the molecular weight of the carrier: (a) radiolabeled monoclonal antibodies (b) receptor specific small proteins and peptides and (c) small molecules.

## 2. Carrier Molecules

**Figure 1:**
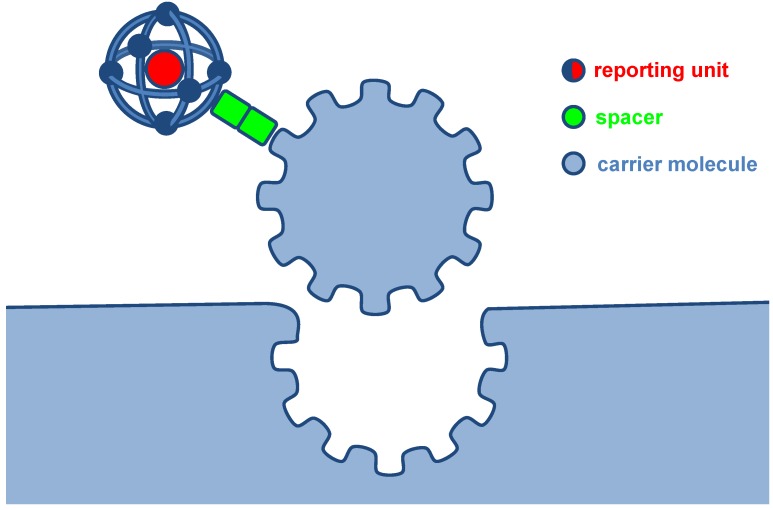
Binding of ligand to target like a peptide-receptor has been visualized by a “lock and key” arrangement, where the peptide fits into a binding pocket of the receptor on the surface of tumor cells in a similar manner to a key fitting into a lock.

Many tumors overexpress specific targets on the surface of their cells. The target ligands are used with radiolabels in cancer diagnosis and therapy in accordance with the key-lock principle (Figure 1). As the number of receptors on the surface of tumor cells compared with that in normal tissues often is higher, the effect on the tumor cells is stronger than that on the normal cells resulting in a wide therapeutic window [6].

### 2.1. Small Molecules

A variety of molecular and functional alterations has been shown to change the morphology and functional status of tumor tissue. Molecular imaging has been established as a tool to measure biomarkers or indicators of disease or therapeutic effects [7]. There are numerous different carriers that have been designed and developed for the targeting of tumors. Several radiolabeled small molecules have been applied *in vivo* for PET imaging [5]. PET radiopharmaceuticals have a significant potential for routine clinical imaging studies. The efficiency of these radiotracers is based on their ability to accumulate in the tumor cells (Table 1).

### 2.2. Antibodies

Antibodies with a very high specificity for their target antigen overexpressed in tumors can display a direct therapeutic effect and must therefore not necessarily be combined with a drug for application as anticancer drugs. However, as many antibodies are not sufficiently cytotoxic, radionuclides have been shown to significantly enhance the therapeutic effects of monoclonal antibodies (mAb). Radiolabeled antibodies exert a certain cytotoxic effect on surrounding cells, depending on the emitted energy of radionuclide radiation over its reach in the tissue decides. In contrast, the unlabeled antibodies interaction is limited on the targeted cells [8]. Zevalin^®^, a ^90^Y-anti-CD20 mAb and Bexxar^®^, a ^131^I-anti-CD20 mAb have been shown to ideally fulfill this task by selectively transporting radionuclides to tumors [9,10].

### 2.3. Peptides

**Figure 2:**
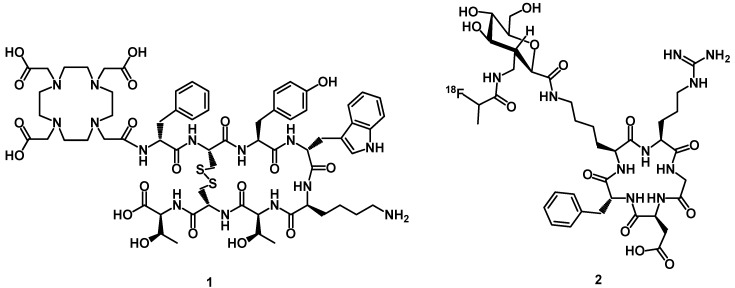
Chemical structures of DOTA-TATE and [^18^F]Galacto-RGD, two typical radiolabeled peptide tracers.

Several receptors with small regulatory peptide ligands are overexpressed in certain human cancers, offering the possibility to target these tumors with radiopeptides. The somatostatin analogs DOTA-TOC and DOTA-TATE (**1**) can be labeled with ^111^In or ^68^Ga for imaging, or with ^90^Y, ^177^Lu for radiotherapy of somatostatin receptor (SSTR)-positive tumors (Figure 2). The excellent results obtained led to the development of analogs of other peptide families, such as bombesin, neurotensin, cholecystokinin/gastrin, exendin, RGD (Arg-Gly-Asp) and substance P. Numerous radiolabeled peptides are currently under preclinical research or clinical evaluation for both diagnostic imaging of peptide receptor expression [11,12] and peptide receptor mediated therapy (PRRT) [13,14,15].

#### 2.3.1. Peptides and Radiopeptides as Targeting Agents

The overexpression of peptide receptors in human tumors led to the development of peptide radio-pharmaceuticals for specific diagnostic imaging and/or therapy of cancers. Table 2 summarizes the receptors-binding peptides and their specificity of overexpression in tumors. Neuroendrocrine tumors (NETs), including primaries and metastases, overexpress somatostatin receptor types (sst1-sst5) [6], particularly sst2 [16]. These receptors present the molecular basis for peptide-based probes for cancer imaging and therapy. The somatostatin analogs DOTA-TOC and DOTA-TATE (1) can be labeled with ^111^In, ^64^Cu and ^67/68^Ga for *in vivo* imaging of SST receptor-expressing tumors [17] or with β-emitters (^90^Y or ^177^Lu) or α-emitters (^213^Bi or ^225^Ac), these labeled analogs can be utilized for peptide receptor mediated therapy (PRRT) [14]. For bombesin receptors family, four subtypes are known (BB1-BB4). Gastrin-releasing peptide receptor (GRPR/BB2) has been found to be overexpressed in a variety of tumors, including prostate, breast, pancreas, gastrointestinal and small cell lung cancer [6]. Several radiolabeled bombesin-like peptides, which bind to BN/GRP receptors with high affinity, have been developed in order to be used for diagnostic and/or therapeutic purposes. Bracco has developed the first radiolabeled BN analog [^177^Lu]-AMBA for imaging and PRRT [18,19]. Bombesin antagonists with favorable tumor-to-normal tissue ratios have been by developed Manci *et al.* [20,21,22]. The preliminary clinical study shows that [^64^Cu]-CB-TE2A-AR-06 is a promising ligand for imaging GRP-Receptor-positive tumors in humans [23]. An other application of peptide-ligands as attractive agents is radiolabeled peptides based on the lead structure cyclo(Arg-Gly-Asp-d-Phe-Val) as the integrin α_v_β_3_-targeted radiotracers. Many radiolabeled cyclic RGD peptide antagonists have been evaluated for imaging integrin α_v_β_3_-positive tumors by SPECT or PET [24,25]. Among the radiotracers evaluated in preclinical tumor-bearing models, [^18^F]Galacto-RGD (**2**) is currently under clinical studies in patients suffering from malignant melanomas, sarcomas, head and neck cancer, glioblastomas, and breast cancer [5]. Cholecystokinin (CCK) receptors have been identified in numerous human cancers, like medullary thyroid carcinomas, small cell lung cancers, stromal ovarian cancers and astrocytomas [6]. Radiolabelled CCK/gastrin analogues have been synthesized and characterized for imaging using positron emission tomography and single photon emission computed tomography imaging. All peptides are mostly based on the C-terminal CCK receptor-binding tetrapeptide sequence Trp-Met-Asp-Phe-NH_2_. ^99m^Tc-demogastrin 2 has been evaluated and compared with [^111^In]-DOTA-CCK8 and [^111^In]-DOTA-MG11 in patients with medullary thyroid cancers (MTC) [26]. The results obtained show that [^99m^Tc]-demogastrin 2 showed the best visualization, which may be due to better imaging properties of ^99m^Tc as compared to ^111^In. The glucagon-like peptide-1 receptor (GLP-1R) is one of the most frequently studied peptide receptors. The high density of glucagon-like peptide-1 receptors (GLP-1R) in human insulinomas provides an attractive target for molecular imaging and internal radiotherapy [6]. For this purpose DTPA- and DOTA-conjugate of exendin-4 were synthesized. The peptide [Lys^40^(Ahx-DOTA)-NH_2_]-Exendin-4 radiolabeled with ^111^In shows success in the detection of tumors in patients with insulinomas [27,28,29]. Using the Auger electrons of ^111^In, [Lys^40^(Ahx-DOTA)-NH_2_]-Exendin-4 was evaluated as a radiotherapeutic for glucagon-like peptide-1 receptor-targeted therapy for insulinoma [30]. The peptide receptors, melanocortin receptors exist in five subtypes. The melanocortin 1 receptor (MC1R) is overexpressed in most murine and human melanoma metastases [6], and hence is an attractive target for the detection and treatment of these cancers. Radiolabeled α-MSH analogs, contain the sequence His-Phe-Arg-Trp. They have been developed for MC1R targeting. Recently data demonstrates that radiolabeled α-MSH analogs DOTA-Nle-CycMSH_hex_ and DOTA-Re-CCMSH(Arg^11^) are potential candidates for diagnostic imaging or radiotherapy of melanoma tumors [31,32]. The overexpression of neurotensin receptor NTR1 has been found in several human cancers including Ewing sarcomas, meningiomas, astrocytomas, medulloblastomas and pancreatic carcinomas [6], and several NT analogs have been synthesized and conjugated with a chelator, like DTPA or DOTA. Among all the radiopeptides, DOTA-NT-20.3 is a promising candidate for ^68^Ga-PET imaging of neurotensin receptor-positive tumors [33]. Human adenocarcinomas of the gastroenteropancreatic system overexpress vasoactive intestinal peptide (VIP) receptors [6] and therefore represent logical diagnostic targets for receptor scintigraphy. ^99m^Tc labeled VIP analog (TP3654) is a promising agent for imaging colorectal cancer [34]. Neurokinin type 1 (NK-1) receptors are overexpressed in malignant gliomas. The radiopeptide [^111^In]/[^90^Y]-DOTAGA-substance P binds to these receptors and can be used for treatment of brain tumors [35]. Neuropeptide Y receptors involve Y1R and/or Y2R have been found to be expressed in neuroblastoma, breast carcinomas, ovarian cancers [6]. The chemokine receptors CXCR4 are highly expressed in breast and prostate cancer. These receptors (NPY1R and CXXR4) are promising additional candidates in the oncology field and their advanced status is under preclinical studies.

**Table 2:** Peptide receptors, disease indications and peptide probe in clinical use.

Peptide	Receptor	Tumor Type	Peptide probe
Somatostatin	sst2	Gastroenteropancreatic neuro-endocrine tumors	DTPA-octreotide/DOTA-TOC/DOTA-TATE
Bombesin	GRPR	Breast, prostate and gastro-intestinal stromal cancer	AMBA/CB-TE2A-AR-06BZH3
RGD	α_v_β_3_	Melanomas	[^18^F]Galacto-RGD
CCK/gastrin	CCK2R	Medullary thyroid carcinomas	[^99m^Tc]-demogastrin 2
GLP-1/exendin	GLP-1R	Insulinomas	[Lys^40^(Ahx-DOTA)-NH_2_]-Exendin-4
α-MSH	MC1R	Melanomas	DOTA-Nle-CycMSH_hex_ DOTA-Re-CCMSH(Arg^11^)
VIP	VIPR	Colorectal cancer	TP3654
substance P	NK-1R	Glioblastoma	DOTAGA-substance P

The fact that various receptor subtypes can be expressed simultaneously on tumors provides the possibility to improve the efficiency of peptide tracers *in vivo* multireceptor targeting. As neuroendrocrine tumors (NETs) usually overexpress somatostatin receptors, enables the use of radiolabeled somatostatin analogues. As other peptide receptors have been found to be overexpessed on certain NETs, they can be targeted for radionuclide therapy and imaging of NETs. Examples are radiolabelled gastrin analogues for MTCs and radiolabelled exendin analogues for insulinomas [17,36,37,38].

In the case of tumors simultaneously expressing several types of receptors, targeting of multireceptor overexpressed tumors can be performed by the use of heterodimeric peptides as molecular imaging agents. Better tumor affinity and pharmacokinetics can be achieved through these multivalent interactions. The development of a heterodimeric RGD-bombesin derivatives such as X-RGD-Glu-6-Ahx-BBN(7-14)-NH_2_ [X = [^18^F]SFB (**11**), DOTA (**28**) and NOTA (**29**)] demonstrated favorable pharmacokinetic properties, resulting in a more specific targeting and higher imaging quality of gastrin-releasing peptide receptor (GRPR) [39,40,41,42]. Josan *et al*. have prepared a peptide heterodimer MSH(7)-CCK-6 that binds to two G protein-coupled receptors: melanocortin-4 (MC4R) and cholecystokinin-2 Receptors (CCK2R) [43]. By using solid-phase synthetic strategy, heterobivalent ligands targeted to melanocortin-4 (MC4R) and δ-opioid (δ-OR) receptors were prepared [44]. The heterodimeric peptides are provided to illustrate the relative enhancement in binding affinity to receptors overexpressed tumor cells.

#### 2.3.2. Characteristics and Challenges of the Synthesis of Peptide-based Radiopharmaceuticals

Peptides are important regulators of growth and cellular functions in normal tissue and tumors. In oncology, major progress has been made with radiolabeled peptide analogs for *in vivo* localization and therapy of tumors. With the advances in organic, bioconjugate and coordination chemistry, solid phase peptide synthesis and phage display techniques radiolabeled peptides with high receptor binding affinity for a selected target have been developed [45]. Generally, peptides offer distinctive advantages over other carriers like small molecules, proteins and antibodies. Peptides, cover many biologically important targets, have high receptor binding affinity, are of relatively low molecular weight, easy to synthesize, accessible to modification like conjugation with chelators for radiolabeling which allows straightforward kit-preparation of peptide radiopharmaceuticals, provide favorable pharmacokinetics resulting in a rapid whole body clearance, good tumor penetration and reach it in high concentration and For therapeutic purposes, they are applied at doses lower than conventional drugs, and therefore cause few side effects and in addition lack immunogenicity [46].

A variety of strategies have been applied to enhance the bioavailability of radiolabeled peptides. The introduction of unnatural or D-amino acids and shortening of the sequence of natural molecules to the biological active sequence are strategies to prolong the biological half-life. A typical example is the optimization of radiolabeled RGD peptide including multimererization for improvement of the binding affinity for the α_v_β_3_ receptor [47]. Pharmacokinetic modifications such as introduction of charged amino acids, glycosylation [48] and PEGylation have also been applied [49].

The radioactive halogens or metals are the most frequently used elements to prepare peptide based radiopharmaceuticals. The radiolabeled peptides provide a class of targeting molecules appropriate for both molecular imaging and radiotherapy. The fact that many metallic radionuclides form stable complexes with similar chelators allows for the labeling of the same peptide or peptide conjugate with various radionuclides for different purposes. The labeling protocols include covalent labeling, either direct or indirect using prosthetic groups, or labeling strategies using bifunctional chelating agents (BFCAs). Ligands that contain two different moieties, a chelating unit to complex the radiometal and a functional group for the covalent attachment of the peptide, are known as bifunctional chelating agents (BFCAs). Prosthetic groups are bifunctional agents that consist of a suitable site for radioiodination or fluorination and functional groups to allow covalent attachment of the peptide. Several of them have been designed and evaluated to engineer high thermodynamically and kinetically stable radiolabeled peptides to prevent the release of the radionuclide. In the following sections, the methods for radiolabeling with clinically relevant radionuclides and the developments of BFCAs, based on polyaminopolycaboxylate, including acyclic and macrocyclic chelator are discussed.

## 3. Label Types

### 3.1. Iodine-Labeled Peptide Radiopharmaceuticals

**Figure 3:**
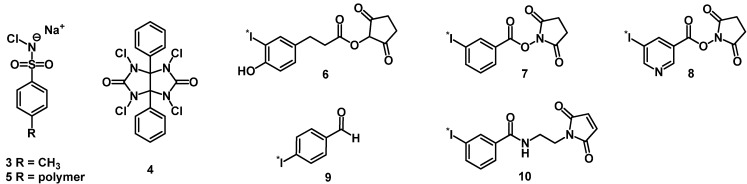
Chemical structure of *N*-chloroamide oxidizing agents and prosthetic groups for radioiodination of peptides.

Generally, radioiodination of peptides can be performed using one of following methods: radioiodination by electrophilic substitution (direct) or radioiodination via conjugation (indirect). The tyrosine or histidine side chains in peptides offer the possibility of electrophilic aromatic substitution by electrophilic radioiodine (*I^+^) with high efficiency under mild conditions. Several oxidizing agents can be used for the generation of electrophilic iodine (*I^+^) such as chloramine T (sodium tosylchloroamide) (**3**) or Iodogen^®^ (1,3,4,6-tetrachloro-3α,6α-diphenylglycoluril) (**4**) (Figure 3). Polymer-bound chloramine T (IodoBeads^®^) (**5**) or vials coated with Iodogen^®^ have the advantage that no reducing agent is needed to quench the labeling reaction, since they are insoluble and can be easily separated from the reaction mixture.

Indirect labeling is another strategy for iodination of peptides, when direct labeling is not possible. The incorporation of radioiodine can be performed by the utilization of radioiodinated prosthetic groups, which can be used for conjugation with specific functionalities introduced previously into the biomolecule or peptide precursors such as amine, aminooxy or thiol groups. Due to the disadvantage of the Bolton-Hunter reagent (*N-*succinimidyl-3-(4-hydroxy,5-[*I]iodophenyl)-propionate) (**6**) of low *in vivo* stability, other active esters have been developed (Figure 3). Among these, SIB (*N-*succinimidyl-3-[*I]iodobenzoate) (**7**) and SIPC (*N-*succinimidyl-5-[*I]iodo-3-pyridine carboxylate) (**8**) are very stable against *in vivo* deiodonation and allow high-yield conjugation with amino groups of peptides [50,51]. Aldehydes, such as 4-[*I]iodobenzaldehyde (**9**), have been used for the coupling of peptides to form stable radiolabeled oximes. This methodology has been proposed for radioiodination of multimeric cyclic RGD peptides [52]. Maleimides allow the chemoselective conjugation to thiols in peptides. A radiolabeled maleimide derivative of **10** has been used for a radioiododestannylation approach followed by conjugation with a Cys-peptide under very mild conditions in one step in high yield [53].

### 3.2. Fluorine-Labeled Peptide Radiopharmaceuticals

**Figure 4:**
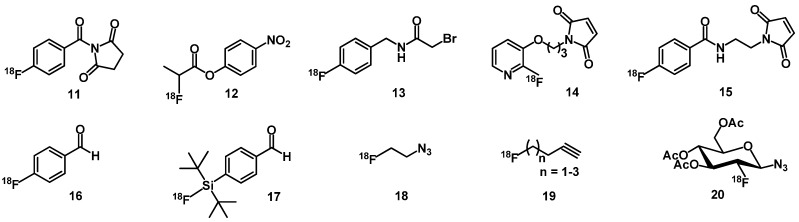
Chemical structure of prosthetic groups for the fluorination of peptides.

^18^F-labeling of peptides by direct labeling is not possible via nucleophilic substitution under mild conditions. Mild conditions are required as the elevated temperatures and strong bases that are used for radiofluorination destroy the peptidic biomolecules. Therefore, ^18^F-labeled prosthetic groups have been developed. For this purpose specific functionalities, such as amine, aminooxy, hydrazine, alkyne or azide groups have to be introduced into the peptide precursor. Amino reactive prosthetic groups (Figure 4) are widely used for [^18^F]fluorination of peptides [24,54,55], since [^18^F]SFB (*N-*succinimidyl-4-[^18^F]fluorobenzoate) (**11**) and [^18^F]NPFP (4-nitrophenyl-2-[^18^F]fluoropropionate) (**12**) allow conjugation in good yield and poses high metabolic stability [56,57]. Numerous [^18^F]fluorinated prosthetic groups based on thiol-maleimide coupling chemistry or thiol-selective alkylation reactions, e.g., *N-*(4-[^18^F]fluorobenzyl)-2-bromoacetamide (**13**), 1-[3-(2-[^18^F]fluoropyridin-3-yloxy)propyl]pyrrole-2,5-dione ([^18^F]FPyMe, **14**) and *N-*2-(4-[^18^F]-fluorobenzamido) ethylmaleimide ([^18^F]FBEM, **15**), have been developed for the conjugating to peptides [58,59]. As the synthesis of these prosthetic groups include multistep procedures, there is still the need for ^18^F-labeling methods suitable for faster peptide labeling. Chemoselective conjugation methods using aldehydes, alkyne or azide derivatives labeled with ^18^F seem to be more efficient for clinical application, such as [^18^F]FBA (4-[^18^F] fluorobenzaldehyde, **16**), [^18^F]SiFA-A (*p-*(di-*t*-butyl[^18^F]fluorosilyl)benzaldehyde) (**17**), [^18^F]fluoro- ethylazide (**18**), [^18^F]fluoroalkynes **19** and [^18^F]-glycosyl azide (**20**) [60,61,62,63,64]. The derivatives are synthesized in one step and used to form oximes, hydrazones or 1,2,3-triazoles of unprotected peptides.

The high lipophilicity of the resulting peptide radiopharmaceuticals derived from the fluorination strategy discussed above leads to a high unspecific liver and low tumour uptake. Glycosylation or polyethylene glycol (PEG) conjugation yields peptides showing lower lipophilicity thus more significantly favorable radiolabeled peptide pharmacokinetics. For example glycosyl-Lys([^18^F]FP)-TOCA and [^18^F]galactosyl-RGD, glycosylated analogs, have been developed [24,65] and evaluated in patients [25,66].

### 3.3. ^99m^Tc-Labeled Peptide Radiopharmaceuticals

**Figure 5:**
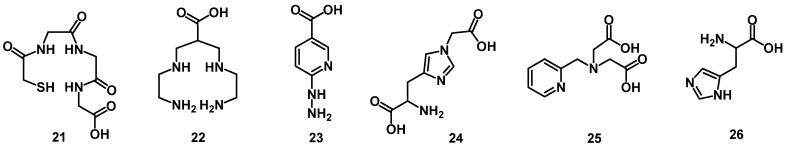
Chemical structure of chelators for the labeling of peptides with ^99m^Tc.

^99m^Tc is still the most frequently used radionuclide in diagnostic applications of nuclear medicine, due to its ideal nuclear physical properties, availability through a commercial ^99^Mo-^99m^Tc generator, the low production cost and easy and rich labeling chemistry. Most radiopharmaceuticals have ^99m^Tc-complexes in the oxidation state of +V. ^99m^Tc is eluted from the generator in physiological saline in its chemically inert oxidation state of +VII as the complex ion ^99m^TcO4^−^. ^99m^Tc(+VII) must be reduced to a lower oxidation. For this purpose different reducing agents, such as Na_2_S_2_O_4_, SnCl_2_, phosphines or zinc, can be used in the presence of suitable ligands. The labeling of peptide based radiopharmaceuticals usually follows the postconjugation labeling strategy. A bifunctional chelator is first covalently bound to the peptide. Subsequently, ^99m^TcO_4_ˉ is reduced with Sn(II) and complexed by the chelator. For this strategy a variety of bifunctional chelators have been designed and tested (Figure 5). The structure of the resulting complexes and the oxidation state of technetium depend on the reducing agent, the ligand as well as coligands. The tetradentate bifunctional chelators based on N_3_S [67,68], N_2_S_2_ [69], such as MAG_3_ (mercaptoacetyltriglycine) (**21**), form square pyramidal complexes containing the [Tc=O]^3+^ core. The *trans*-[O=Tc=O]^+^ core, which forms octahedral complexes can be prepared with tetraamine ligands [70,71,72,73,74,75,76]. The use of N_4_ cores (**22**) offers the advantages of hydrophilic Tc-complex without isomeric structural influence. HYNIC (hydrazinonicotinic acid, **23**) is widely used for the coupling of technetium to peptides [77,78,79]. It acts as a mono or bidentate ligand [80]. In both cases coligands, such as EDDA, tricine or nicotinic acid are required to complete the coordination of the [Tc]-HYNIC core. The use of coligands can have a positive side effect, since they can influence the lipophilicity of the radiopharmaceutical and the *in vivo* stability of the ^99m^Tc-complexes, which in turn affects the radiopeptide pharmacokinetics [81].

Another strategy is the labeling of biomolecules with the organometallic [^99m^Tc(CO)_3_]^+^ core. The ^99m^Tc tricarbonyl approach has been used for the development of new radiopharmaceuticals with the organometallic precursors fac-[^99m^Tc(CO)_3_(H_2_O)_3_]^+^. ^99m^Tc-tricarbonyl complexes which are formed with a tridentate BFC conjugated peptide, such as (Nα-His)Ac (**24**) or picolylamine diacetic acide (PADA) (**25**) show better stability *in vivo*, compared to mono and bidentate ligands such as histidine (**26**) [82]. Finally, the application of HYNIC and the N_4_-approach for peptide conjugation results in products with highly favorable pharmacokinetics in animal models and patients [12]. 

### 3.4. ^111^In/^67/68^Ga/^86/90^Y/^177^Lu/^64/67^Cu-Labeled Peptide Radiopharmaceuticals

**Figure 6:**
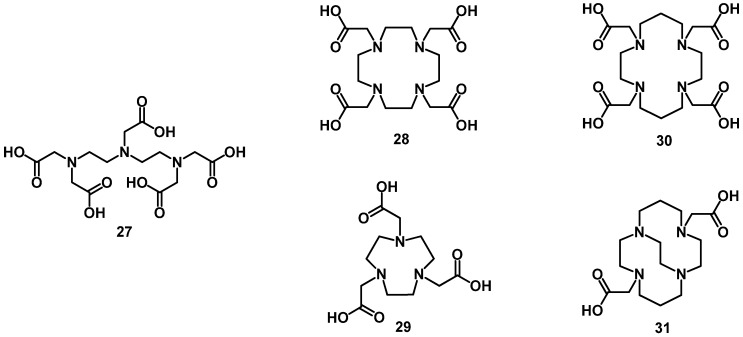
Chemical structures of chelators that are suited for the labeling of peptides with radiometals such as ^111^In/^67/68^Ga/^86/90^Y/^177^Lu/^64/67^Cu.

Both Ga^3+^ and In^3+^ are hard Lewis acids, because of their high charge density and small ionic radius. For this reason, hard ligands form thermodynamically stable complexes with these ions (HSAB concept). These ligands contain nitrogen and oxygen donor atoms. In^3+^ is softer and larger than Ga^3+^. This difference often leads to a different coordination chemistry. The first clinically established peptide radiopharmaceutical for the visualization of neuroendocrine tumors was DTPA-octreotide labeled with ^111^In (under the name of Octreoscan^®^) [83]. DTPA (diethylenetriaminepentaacetic acid, **27**, Figure 6) is still one of the choices of BFCs for the labeling of peptides with ^111^In. It forms stable ^111^In-complexes with fast labeling kinetic. However, it does not suit well for the labeling with many of the clinically used β-emitters such as lanthanides [84]. The macrocyclic chelator DOTA (1,4,7,10-tetraazacyclododecane-1,4,7,10-tetraacetic acid, **28**, Figure 6) has been evaluated for the labeling of peptides with divalent and trivalent radiometals, such as Ga^3+^, In^3+^, Y^3+^, Lu^3+^ and Cu^2+^. It forms thermodynamically and kinetically stable complexes. Several DOTA-peptide conjugates labeled with gallium and indium have been used in clinical routine like the somatostatin conjugates DOTA-TOC and DOTA-TATE, bombesin analogs, RGD analogs, minigastrin analogs *etc.* The macrocyclic chelator NOTA (1,4,7-triazacyclononane-1,4,7-triacetic acid, **29**, Figure 6) is most favorable for the ^67/68^Ga-labeling of peptides [85]. The difference in cavity size of NOTA and DOTA is another important aspect to consider when selecting a proper chelator for the labeling with radiometalls. The thermodynamic stability constant of Ga-NOTA complex is approximately 10 orders higher than that of Ga-DOTA [86]. NOTA derivatives containing an additional coupling moiety that was introduced into the macrocycle such as benzyl-isothiocyanate (NOTA-Bz-NCS) or at the α-position of one carboxylate arm such as aspartic acid (NODASA), glutamic acid (NODAGA) and benzyl-isothiocyanate (NODAPA-NCS) have been developed. The advantage of this additional coupling moity is that all of the carboxylic arms are available to saturate the hexadentate coordination.

Yttrium and lanthanide ions with an oxidation state of 3^+^ are hard Lewis acids as well, and tend to form very stable complexes with hard ligands. Because of their large size, their complexes have the high coordination numbers of 8 and even 9. The labeling of peptides with these radionuclides has been performed using mainly macrocyclic polyaminopolycarboxlic bifunctional chelating agents [87]. Due to the favorable pharmacokinetic profiles, DOTA derivatives have been used for the ^86/90^Y and ^177^Lu labeling of various peptides. DOTA provides eight donor atoms and the appropriate cavity size to form more stable complexes with these radionuclides than acyclic chelating agents and TETA (1,4,8,11-tetraazacyclododecane-1,4,8,11-tetraacetic acid, **30**, Figure 6) derivatives. Thermodynamic stability and kinetic inertness are the most important factors for *in vivo* applications. DOTA derivatives were efficiently coupled to peptides as DOTA monoamides, where the kinetic inertness *in vivo* is not changed in comparison to DOTA. DOTA and TETA have been used for the production of ^67/64^Cu-labeled peptides. They show moderate kinetic stability under *in vivo* conditions. Cu-complexes with cross-bridged cyclam such as CB-TE2A (4,11-bis(carboxymethyl)-1,4,8,11-tetraazabicyclo[6.6.2]-hexadecane, **31**, Figure 6) improve the kinetic inertness and thus the pharmacokinetics of the radiolabeled peptide.

## 4. Chelator Types

### 4.1. Acyclic Chelators

**Figure 7:**
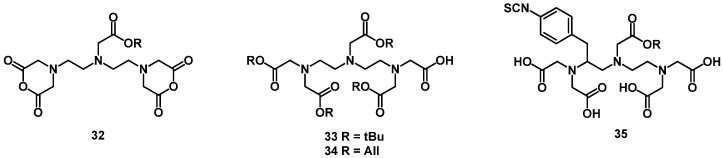
The chemical structure of BFCAs derived from DTPA.

**Scheme 1:**
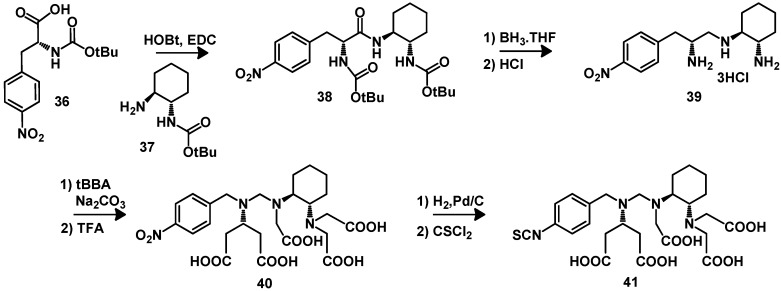
Synthesis of CHX-A''-DTPA.

[^111^In]-DTPA-octreotide (OctreoScan^®^) is an octreotide derived somatostatin analog known to act as a selective molecular targeting agent for the imaging of neuroendocrine tumors. It has been widely used as imaging agent in single photon emission computer tomography (SPECT). DTPA (diethylenetriaminopentaacetic acid, **27**) was first synthesized by Frost [88]. DTPA and its derivatives (Figure 7) can be used for the complexation of radiometals like ^111^In, ^213^Bi, ^86/90^Y, ^177^Lu, ^99m^Tc, ^67/68^Ga for nuclear medicine applications and for Gd for MRT applications. The conjugation of DATP using its reactive mixed anhydride [89] or cyclic bisanhydride **32** [90] can lead to undesired conjugates, in particular double substituted DTPA side products [91]. To avoid this drawback, DTPA-tetra(*t-*Bu ester) (**33**) or DTPA-tetra(All ester) (**34**) have been synthesized starting from diethylenetriamine [92,93]. The activation of the free carboxylic group with activation agents like DIC or HBTU allows the defined conjugation with primary amines of proteins or peptides. A major advantage of using **33** is its commercial availability and that it readily reacts in solid phase synthesis reactions. In addition, this BFCA shows a fast labeling kinetic and thus the reaction conditions for radiometallation can be applied for sensitive biomolecules. Unfortunately, in contrast to the radionuclide ^111^In, most of the commonly applied β-emitters do not form complexes with sufficient kinetic stability for radiotherapy [84]. DTPA derivatives bearing a functional isothiocyanate group like **35** were synthesized to allow selective conjugation of an amino group of a protein under mild reaction conditions. In these conjugates all carboxylic arms are available for coordination to the radiometal. The geometry of the coordination sphere can be optimized by introducing a preorganizing group on the DTPA backbone [94,95,96]. The ligands obtained can be conjugated to biomolecules and form radionuclide complexes with greater kinetic stability *in vivo* and faster complexation kinetic. These DTPA derivatives are viable alternatives to DOTA for radiometallation of monoclonal antibodies [97,98,99] or for labeling with short half-live isotopes like bismuth-212 and bismuth-213 [97]. CHX-A''-DTPA (**41**) has to be considered as a suitable BFCA for the radiolabeling of monoclonal antibodies with β-emitters for radioimmunotherapeutical applications. Scheme 1 includes the synthesis of CHX-A''-DTPA. The first FDA-approved therapeutic agent for treatment of patients with lymphoma was ibritumomab tiuxetan (Zevalin^®^, Cell Therapeutics Inc, Seattle, WA, and Schering AG, Berlin, Germany). It is labeled with ^90^Y using an acyclic DTPA based BFCA.

**Scheme 2:**
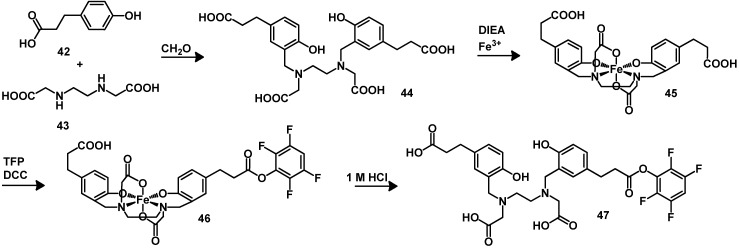
Synthesis of activated ester of HBED-CC.

Among the variety of potential acyclic BFCAs, HBED (*N,N′*-bis[2-hydroxybenzyl]ethylenediamine-*N,N′*-diacetic acid)-based ligands have been used for the radiometallation of biomolecules [100,101]. HBED-CC (*N,N′*-bis[2-hydroxy-5-carboxylethyl-benzyl]ethylenediamine-*N,N′*-diacetic acid, **44**) forms complexes with ^68^Ga with high *in vivo* stability. The use of HBED-CC as BFCA is similar to the method for producing ^68^Ga-radiolabeled proteins with fast complexation kinetics at ambient temperature that can be used as diagnostic agent, e.g., for positron emission tomography (PET) imaging [102]. For this aim the (HBED-CC)TFP active ester **47** has been described and synthesized by activating one of the two carboxyl groups of the [Fe(HBED-CC)]−complex with DCC and tetrafluorophenol in DMF. The demetallation can be performed with 1 M HCl (Scheme 2).

When coupled to the PSMA-specific pharmacophore Glu-NH-CO-NH-Lys HBED-CC has been shown to specifically bind and enter into prostate cancer cells [103]. The PET/CT images of prostate cancer obtained with ^68^Ga-labeled HBED-CC-Ahx-Lys-NH-CO-NH-Glu are superior to the imaging agent [^18^F]FECH [104].

**Scheme 3:**
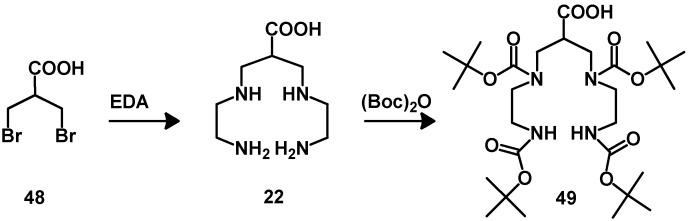
Synthesis of the N_4_-chelator.

Acyclic BFCAs have also been used for the labeling of biomolecules with ^99m^Tc. Recently, it was found that tetraamine-based ligands show superiority to all the other chelators for ^99m^Tc, with whom the labeling reaction can be performed at RT. They were also shown to form central and hydrophilic ^99m^Tc-complex with high kinetic inertness, as well as improved pharmacokinetic including fast blood clearance and specific tumor uptake [70,71,72,73,74,75]. The synthesis of the N_4_-chelator **49** has been facilitated by the reaction of 3-bromo-2(bromoethyl)propionic acid with ethylenediamine (Scheme 3). The protection of the resulting intermediate with Boc protecting groups allows the conjugation with primary amines of peptides on solid phase [76].

### 4.2. Macrocyclic Chelators

Macrocyclic chelators offer the advantage, over the corresponding acyclic chelators, that their radiometal complexes are more thermodynamically stable and remarkably more kinetically inert to dissociation (macrocyclic effect). The introduction of cyclic polyaminopolycarboxylic ligands as BFCAs , such as DOTA, NOTA, TETA and CB-TE2A for the radiometallation of peptides with Ga^3+^, In^3+^, Y^3+^, Lu^3+^ and Cu^2+^ improve the pharmacokinetics of radiophamaceuticals [105].

#### 4.2.1. DOTA and TETA

**Scheme 4:**
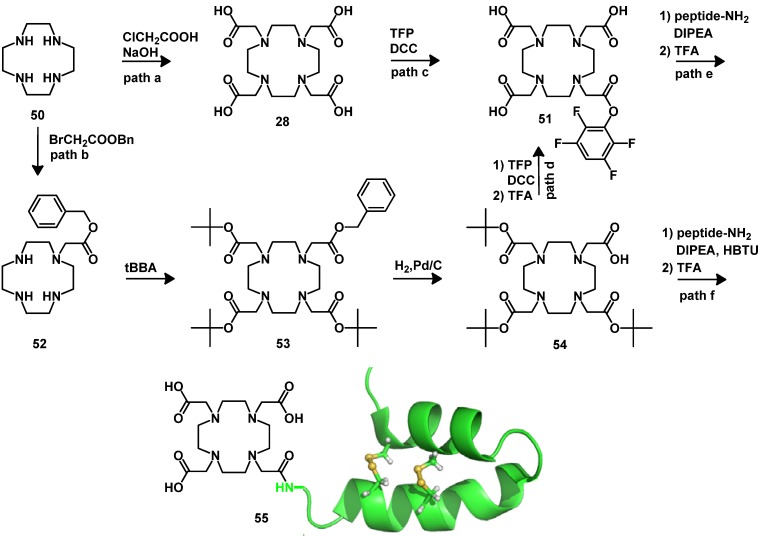
Synthesis of DOTA, DOTA active ester and DOTA-tris(*t-*Bu ester) and their coupling with peptides.

The synthesis of DOTA first reported by Stetter and Frank in 1976 [106] involved the reaction of cyclen **50** with chloroacetic acid under aqueous alkaline conditions (Scheme 4, path a). DOTA (**28**) is the most efficient chelator for both diagnostic and therapeutic applications in nuclear medicine, due to the high kinetic stability of its complexes. In oncology, major progress has been achieved using DOTA-ligands for the preparation of contrast agents for magnetic resonance imaging (MRI). In addition, the contrast agents based on macrocyclic chelators ensure that the Gd-complexes are, when compared to the corresponding complex of DTPA, kinetically more inert. DOTA also is the favorable BFCA for the preparation of therapeutic lanthanide radiopharmaceuticals [91]. Besides, it forms very stable complexes with divalent and trivalent radionuclides like ^67/68^Ga, ^111^In, ^86/90^Y and ^64/67^Cu [86,107,108,109,110,111,112,113]. Several different species of DOTA-based bifunctional chelators have been described for attaching the DOTA-unit to biomolecules: protected DOTA forms, active DOTA esters and DOTA-derivatives with a coupling moiety that was introduced into the macrocycle or at the α-position of one carboxylate arm. Due to the high efficiency of the conjugation methods available protected forms and active esters are suitable for use in solid phase peptide synthesis. The use of these synthons leads to DOTA monoamides for the coordination of radiometals. This leads to a decreased thermodynamic stability constant, but the kinetic inertness *in vivo* is not changed compared to DOTA. 

DOTA active esters have been established for the labeling of proteins. The approach is based on the activation of one of the carboxylic groups. Different activated DOTA esters have been synthesized to optimize the synthesis of DOTA-conjugated biomolecules: (a) mixed anhydride derivative which was prepared using isobutyl chloroformate in the presence of TMG [89]; (b) the active ester DOTA-NHS obtained by direct activation with NHS and EDCI in a water-DMF mixture and followed conjugation to protected peptide [114] and (c) DOTA-phenolic active esters [115]. The class (c) constitutes the favorable precursors, allowing selective conjugation in high yields without the formation of double substituted DOTA side products. In addition, the phenolic esters can be varied that the hydrolytic stability is adopted for the respective application. DOTA-phenolic active esters, such as DOTA-TFP (**51**), can be prepared starting from DOTA or DOTA-tris(*t-*Bu ester) (**54**) by esterification of one carboxylic group using DCC or DIC and the corresponding phenol in a water-acetonitrile mixture (Scheme 4, paths c and d), offering a BFCAs with comparable reactivity to BOP- and HBTU-style reagents [116] which can react with amino groups of peptides or proteins (Scheme 4, path e).

The alternative approach is to use protected DOTA derivatives. For solid phase peptide synthesis it is commonly introduced in its protected tris(*t-*Bu ester). However, the cleavage of the *t-*Bu protecting groups of DOTA-tris(*t-*Bu ester) (**54**) is known to be sluggish [117]. In the solid phase peptide synthesis process it is best performed by the successive treatment with TFA/radical scavenger cocktails followed by reaction with neat TFA [118]. In the case of DOTA-tris(*t-*Bu ester) incomplete deprotection of the *t-*Bu groups often leads to significantly reduced yields and as consequence to complicated purification steps. Several attempts have been made to synthesize DOTA with protecting groups that can be removed under mild conditions such as DOTA-tris(allyl ester) [119], DOTA-tris(methyl ester) [120] and DOTA-tris(benzyl ester) [121]. However, as these methods are either complicated or not convergent to the solid phase peptide synthesis process. These derivatives have not yet found widespread application. As an alternative, the DOTA-tris(OPp ester) has been recently reported [122]. This protecting group is orthogonal to Fmoc and convergently cleaved under the cleavage conditions of the standard amino acid protecting groups.

**Scheme 5:**
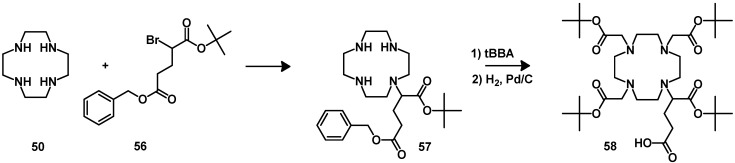
Synthesis of DOTAGA.

Different synthetic approaches have been followed to improve the synthesis of DOTA-tris(*t-*Bu ester) (**54**), the synthesis of DO3A-tris(*t-*Bu ester) [123,124] and the total solid-phase synthesis of the DOTA chelator on solid support [125]. The synthesis can be achieved by the procedure described by Mäcke [126,127] (Scheme 4, path b). This strategy is still widely used for the synthesis of several other structurally related BFCAs. It starts from commercially available cyclen (**50**). The first step of the synthesis involves the monoalkylation of an orthogonally protected alkyl bromoacetate. After full *N-*alkylation and orthogonal deprotection of one of the carboxylic groups, the remote carboxylic acid can be activated using standard coupling agents, such as HATU, and subsequently coupled to amino groups of peptides on solid support (Scheme 4, path f). The same strategy has been followed for the synthesis of DOTA derivates bearing a coupling moiety at the α-position of one of the carboxylate groups. DOTAGA (**58**) was synthesized by Mäcke *et al.* (Scheme 5) [126]. It is compatible with solid phase peptide synthesis. Its synthesis includes the monoalkylation of one amine function with 2-bromo-glutaric-1-*tert*-butyl-5-benzylester (**56**) and then full *N-*alkylation with *tert*-butyl bromoacetate (*t*-BBA). The protecting benzyl group is then removed by hydrogenation with a Pd/C catalyst.

**Scheme 6:**
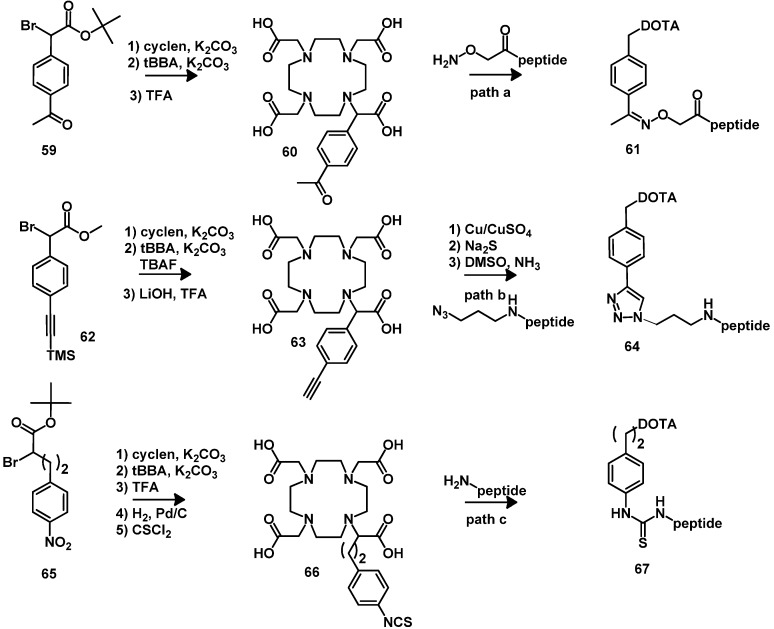
Synthesis of DOTA-derivatives for chemoselective conjugation to peptides.

Knör *et al*. have designed DOTA derivatives **60** and **63** enabling the chemoselective attachment to unprotected functionalized peptides [118]. Cyclen was monoalkylated with *tert*-butyl 2-bromo-2-(4-acetylphenyl)-acetate (**59**) and methyl-2-bromo-2-(4-(2-(trimethylsilyl)ethynyl)phenyl)-acetate (**62**). After full *N-*alkylation with *t*-BBA, the product was deprotected with a standard deprotection mixture of TFA, triisopropylsilane (TIPS) and water (95:2.5:2.5). The obtained BFCAs were directly used for oxime ligation (Scheme 6, path a) or Cu^I^-catalyzed azide–alkyne cycloaddition (Scheme 6, path b) of unprotected peptides. 

**Scheme 7:**
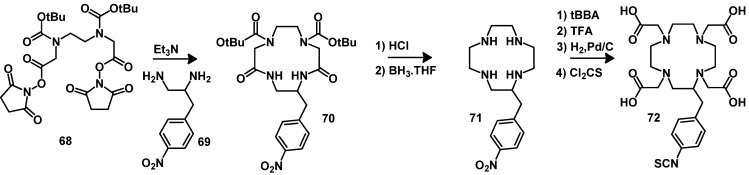
Synthesis of DOTA bearing benzyl-isothiocyanate.

Another important BFCA is **66** (DOTA with an isothiocyanatophenylmethyl coupling moiety). The compound was synthesized starting from cyclen (Scheme 6) [128]. After full *N-*alkylation and deprotection of the *t-*Bu carboxylate protecting groups the nitro group was reduced to an amino group which was then converted to the isothiocyanate group with thiophosgene. The ligand **72**, which is functionalized on a macrocyclic ring carbon atom was described in 1988 [129], its synthesis was optimized in 1992 [130] by reaction of 4-(nitrobenzyl)-ethylenediamine (**69**) with the carbamate protected amino disuccinimido ester **68**. The resulting intermediate **70** was deprotected and its amide functions reduced. Followed by full alkylation with *t*-BBA. The nitro group was further reduced to an amino group which was then converted to the isothiocyanate group with thiophosgene (Scheme 7). The compounds **66** and **72** are commercially available and can be selectively coupled to amino groups of lysine of peptides or proteins under mild basic conditions of pH 8-9 (Scheme 6, path c).

**Figure 8:**
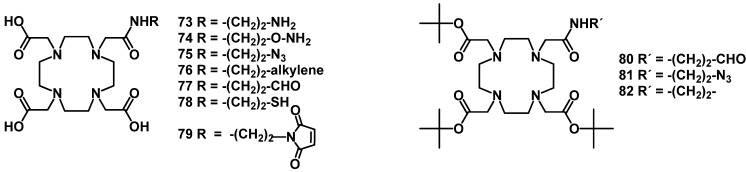
Chemical structures of DOTA-monoamide derivatives for the chemoselective conjugation to peptides.

The DOTA-monoamide (DOTAMA) ligands **73**–**82** bearing amino, thiol, aldehyde, azide and maleimido groups have been described [131,132], which allow chemoselective coupling to suitable groups of biomolecules (Figure 8).

**Scheme 8:**
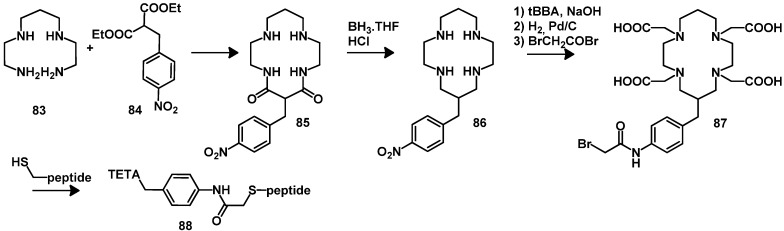
Synthesis of TETA bearing benzyl-isothiocyanate.

The malonic ester **84** has been used to produce a BFCA derived from TETA bearing the coupling moiety benzyl-isothiocyanate [133]. The synthesis includes the reaction between diethyl (*p-*nitrobenzy1)malonate (**84**) with *N,N′*-bis(2-aminoethy1)-1,3-propanediamine (**83**), yielding the macrocycle containing two amide bonds, which can be reduced and alkylated with bromoacetic acid. The conversion of the nitro into an amino group by reduction and by the reaction with BrCH_2_COBr yields BFCA (**87**) which can react with cysteines of peptides or proteins (Scheme 8) [134].

**Figure 9:**
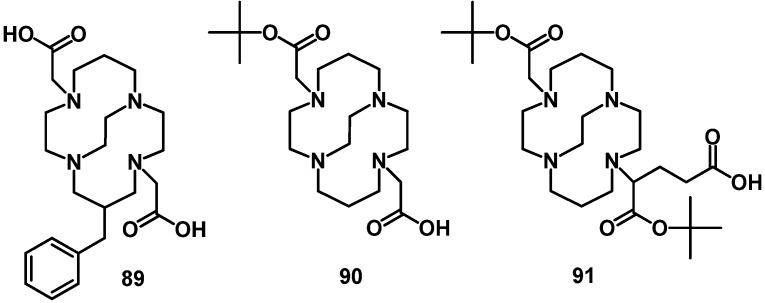
Chemical structures of the CB-TE2A derivatives.

DOTA and TETA have been used for the production of ^67/64^Cu-labeled peptides. However, the liver uptake and blood concentration are high for Cu complexes of DOTA [135] and TETA [136] at all time points, indicating an only moderate kinetic stability under *in vivo* conditions. For this reason efforts have been undertaken to develop BFCAs such as the CB-TE2A derivatives (Figure 9) [137,138] that reduce *in vivo* dissociation and transmetallation reactions. Peptide conjugates containing the BFCAs as cross-bridged, cyclam-based ligands have shown good pharmacokinetics as targeting vectors in comparison to conventional chelators, such as DOTA and TETA [139]. NODAGA is the optimal BFCA for the labeling of peptides with ^64^Cu and ^68^Ga, since their ^64^Cu- and ^68^Ga-labeled conjugates show improved PET imaging properties [139,140] and the labeling can be performed at room temperature with both radionuclides within 10 min in high purity [140].

#### 4.2.2. NOTA

**Scheme 9:**
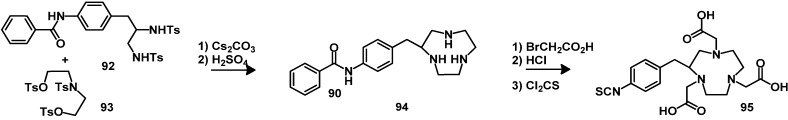
Synthesis of NOTA-Bz-NCS.

The moderate stability of some DOTA radiometal complexes with ions such as ^67/68^Ga and ^64/67^Cu complexes limits the efficiency and application of DOTA for the preparation of ^67/68^Ga and ^64/67^Cu radiopharmaceuticals. The BFCA NOTA has been described in several cases as the optimal chelator for ^67/68^Ga and ^64/67^Cu [85,141]. But only a limited number of publications have described NOTA as BFCA, due to the requirement of a large excess of NOTA for a successful coupling to peptides [135]. NOTA can also be attached to peptides on solid support in a multiple step de novo synthesis [142,143]. Easier conjugation is possible with bifunctional NOTA-ligands. Their synthesis is based on the introduction of a coupling moiety at the α-position of one carboxylate arm such as aspartic acid (NODASA), glutamic acid (NODAGA) or benzyl-isothiocyanate (NODAPA-NCS). In addition the use of these prochelators does not require any heating for ^67/68^Ga-labeling — in contrast to the DOTA-conjugates, where the intensive heating required to overcome the slow labeling kinetic would destroy the proteins. For this reason the preparation of NOTA using selective reactivity of isothiocyanates offer advantages for NOTA-coupling to proteins. Tow different classes have been described: *N*-substituted NOTA derivatives [144] and C-functionalized derivatives of NOTA [145]. They are water soluble and selectively react with the amino side chain of lysine. The Richman-Atkins method has been used for the preparation of a C-functionalized triazamacrocycle 2-(*p-*NCS-Bz)-TACN [146], which was converted to 2-(*p-*NCS-Bz)-NOTA (**95**) by alkylation with bromoacetic acid followed by hydrolysis and reaction with thiophosgene (Scheme 9).

**Scheme 10:**
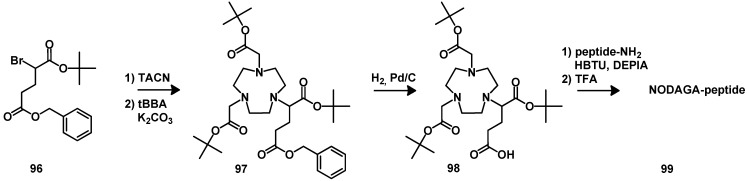
Synthesis of NODAGA.

NODAGA-tris(*t-*Bu ester) (**98**) is useful for coupling to peptides, particularly on solid phase [147]. The synthesis was performed by monoalkylation of the orthogonal protected dicarboxylic acid **96**. After *N*-alkylation with *tert*-butyl bromoacetate the hydrogenation with Pd/C allows the direct activation of the free carboxylic acid and subsequent coupling of the deprotected chelator to the desired peptides. The cleavage and full deprotection are achieved by the treatment with standard TFA peptide cleavage conditions (Scheme 10).

## 5. Synthesis of Chelator Peptide Conjugates

Postconjugation labeling represents the most commonly used labeling strategy. In this strategy a protected peptide is first synthesized on solid phase. Subsequently, a BFCA is conjugated to the resin bound peptide. For this strategy there exist many BFCAs which can be classified into two species: the protected form and active esters. The monoreactive chelators of DOTA derivatives have been shown to be efficiently coupled to resin-bound peptides and then deprotected to DOTA-peptide conjugates under the standard TFA peptide cleavage conditions [118]. DOTA in its protected form tris(*t-*Bu ester) is commercially available and is frequently used for the synthesis of peptide derivatives, especially for the conjugation to peptides on solid phase. The conjugation can be performed by coupling to the *N*-terminal amino group of the peptide chain attached to the resin or to the amino group of a lysine side chain after orthogonal deprotection. To overcome the problem caused by incomplete deprotection of the commonly used protecting groups Mtt, ivDde, or Alloc [148] on the side chain of Lysine, DOTA-coupled amino acid derivatives, such as Fmoc-Lys(DOTA-tris(*t-*Bu ester))-OH and Fmoc-Phe(DOTA-tris(*t-*Bu ester)-NH-)-OH, were synthesized [149,150]. These compounds are fully compatible with solid phase peptide chemistry, and they can be used to introduce the DOTA-unit into any position within the resin-attached peptide sequence. A good example for the synthesis of a DOTA-peptide conjugate is the somatostatin derivative DOTA-TATE (Figure 1). Several protecting group strategies have been used to synthesize the peptide including DOTA-coupling as well as disulfide bridge formation. Generally, the DOTA coupling is followed by a cyclization step on the resin or in solution. Peptides comprising several disulfide bridges are engineered for both molecular imaging and endoradiotherapy. The synthesis of miniproteins has been performed using the Cys(Ypro)/Cys(Trt)/Cys(Acm) protecting group strategy. For the labeling with radiometals, DOTA-DFP is coupled to the amino group of the lysine side chain of the *N-*acetylated Min-23 peptide following the oxidative steps required to form the disulfide bonds [151].

## 6. Conclusions

Postconjugation labeling is frequently used for the labeling of peptides with clinically used radionuclides for imaging and therapy. Many BFCAs have been synthesized and exploited in the area of peptide-based radiopharmaceuticals. For this strategy there exist three classes of BFCAs: the protected forms, active esters and derivatives with a coupling moiety. The most commonly used BFCAs are based on polyaminopolycarboxylic chelating cages, such as DTPA, DOTA, NOTA and TETA. The review discusses the most important BFCAs and their synthetic strategies that allow the preparation of radiopeptides for tumor receptor targeting. Some of them show excellent result in clinical applications such as DOTA-TATE.
